# Interplay between Epstein-Barr virus infection and environmental xenobiotic exposure in cancer

**DOI:** 10.1186/s13027-021-00391-2

**Published:** 2021-06-30

**Authors:** Francisco Aguayo, Enrique Boccardo, Alejandro Corvalán, Gloria M. Calaf, Rancés Blanco

**Affiliations:** 1 Universidad de Tarapacá, 1000000 Arica, Chile; 2 Laboratory of Oncovirology, Department of Microbiology, Instituto de Ciências Biomédicas, Universidade de São Paulo, São Paulo, Brazil; 3 Advanced Center for Chronic Diseases (ACCDiS), Pontificia Universidad Católica de Chile, Santiago, Chile; 4 Instituto de Alta Investigación, Universidad de Tarapacá, 1000000 Arica, Chile; 5 Center for Radiological Research, Columbia University Medical Center, New York, NY 10032 USA; 6 Laboratorio de Oncovirología, Programa de Virología, Instituto de Ciencias Biomédicas (ICBM), Facultad de Medicina, Universidad de Chile, Santiago, Chile

**Keywords:** Epstein-Barr virus, environmental, cancer

## Abstract

Epstein-Barr virus (EBV) is a herpesvirus associated with lymphoid and epithelial malignancies. Both B cells and epithelial cells are susceptible and permissive to EBV infection. However, considering that 90% of the human population is persistently EBV-infected, with a minority of them developing cancer, additional factors are necessary for tumor development. Xenobiotics such as tobacco smoke (TS) components, pollutants, pesticides, and food chemicals have been suggested as cofactors involved in EBV-associated cancers. In this review, the suggested mechanisms by which xenobiotics cooperate with EBV for carcinogenesis are discussed. Additionally, a model is proposed in which xenobiotics, which promote oxidative stress (OS) and DNA damage, regulate EBV replication, promoting either the maintenance of viral genomes or lytic activation, ultimately leading to cancer. Interactions between EBV and xenobiotics represent an opportunity to identify mechanisms by which this virus is involved in carcinogenesis and may, in turn, suggest both prevention and control strategies for EBV-associated cancers.

## Introduction

Approximately 13% of the cancer burden worldwide is etiologically related to viral infections with variations depending on sociodemographic factors [1, 2]. The long-term persistence of viral genomes in the host is a requisite for virus-driven cancer [3, 4]. To date, viruses from only six families are recognized as carcinogens in human beings. These includes several types of human papillomavirus (HPV, *Papillomaviridae*), two members of the *Herpesviridae* family, Epstein-Barr virus (EBV; *Herpesviridae*) and human herpesvirus 8 (HHV-8), Hepatitis B virus (HBV, *Hepadnavidae*), the flavivirus Hepatitis C virus (HCV), Merkel cell polyomavirus (MCPV, *Polyomaviridae*), and the retroviruses human T lymphotropic virus 1 (HTLV-1) and human immunodeficiency virus 1 (HIV). EBV, discovered in 1964, is a DNA virus that persistently infects approximately 90% of the world population [5]. This virus establishes latent persistent infections in B cells and is transmitted via nasopharyngeal secretions [6]. Although primary infection in children is asymptomatic in most cases [7], 50% of those who experience primary infection in early adulthood develop symptomatic infectious mononucleosis (IM) [8]. Different studies revealed that EBV causes several types of neoplasms. Infection with this virus has been associated with Hodgkin’s lymphoma (HL), extranodal natural killer/T lymphocytes (NK/T) cell lymphoma, and other lymphoproliferative disorders [6]. It has also been established that EBV is associated with epithelial tumors, such as nasopharyngeal (NPC) and gastric carcinomas (GC) [9, 10]. Additionally, EBV has been detected in a proportion of oral, breast and cervical cancers, although the etiological role of the virus in these malignancies is, at present, controversial [11]. While most of the human population is persistently infected by EBV, only a small proportion of subjects finally develop EBV-associated tumors, suggesting that additional factors are necessary for the development of disease. Early studies reported that some environmental contaminant xenobiotics can cooperate with EBV to induce peripheral blood lymphocyte (PBL) transformation with increased EBV genome amplification in carcinogen-treated cells [12]. During the last decades, results from different studies established that xenobiotics such as tobacco smoke (TS) compounds, pollutants, food chemicals, and pesticides, among others, may be involved in EBV-associated cancers. Oxidative stress (OS) promoted by some xenobiotics alters EBV gene expression profile and host interactions, both involved in cancer. In this review, we present epidemiological and experimental evidence addressing the interplay between xenobiotics and EBV for carcinogenesis. Additionally, we suggest general mechanisms by which xenobiotics can modulate the EBV replicative cycle during epithelial or lymphoid carcinogenesis.

## Epstein-Barr virus: structure and replicative cycle

Epstein-Barr virus (EBV), also known as human herpesvirus 4 (HHV-4), is a gamma-herpesvirus of the *Lymphocryptovirus* (LCV) genus [6]. The virus is approximately 122 to 180 nm in diameter with a genome composed of a linear double-stranded DNA of approximately 172,000 base pairs packed into an icosahedral capsid containing 162 capsomers. The major capsid protein is the Viral Capsid Antigen (VCA) and the minor capsid proteins are BDLF1 and BORF1 [13]. In addition, the tegument structure, located between the capsid and envelope, includes at least 17 viral proteins needed during the early stages of infection [14]. The envelope is composed of 12 glycoproteins although the fusion machinery is formed by the glycoprotein B (gB), the heterodimeric complex gH/gL, and non-conserved receptor-binding proteins [14]. EBV infection is especially targeted to B cells and epithelial cells. In fact, the EBV replicative cycle has been extensively studied in B cells where the virus establishes latency. Eventually, under conditions of B cell differentiation to plasma cells, it can be reactivated with viral maturation, releasing, and cell lysis.

The EBV replicative cycle in epithelial cells is less understood, at least in part due to the historical absence of an *in vitro* model for efficient viral replication, although a system based on organotypic epithelial cell cultures for EBV replication was recently established [15–17]. Using this model, it was demonstrated that EBV replicates and induces cytopathic effect in oral keratinocytes from the suprabasal layers of stratified epithelium. Cells sustaining EBV amplification expressed both productive-cycle proteins and latency-associated proteins. However, no cells expressing exclusively latency genes were observed [16]. Indeed, latent EBV infection is uncommon and rarely found in normal tissues such as primary nasopharyngeal and oral epithelial cells where EBV only establishes a lytic cycle [18–20]. Interestingly, latently infected epithelial cells are detected in tonsil explants in the presence of acyclovir, albeit in less than 0.01% of cells [21].

Although EBV entry in B cells occurs through endocytosis and subsequent membrane fusion [22], EBV entry in epithelial cells is achieved through direct fusion with the cell membrane, which is carried out by the core fusion machinery composed of the gB and the receptor-binding complex gH/gL [22]. Furthermore, gp350 is required for efficient EBV attachment through the binding to complement receptor type 2 (CR2/CD21), which is expressed in both B cells and tonsillar epithelial cells [23]. The gp42 viral protein is important for B cell entry, therefore the level of this protein in the virion determines EBV tropism [24, 25]. The exact mechanism and receptors involved in epithelial cell entry have remained elusive, although members of the integrin family of proteins, such as αvβ5 or αvβ6, have been suggested as epithelial receptors [26]. However, blocking these integrins does not completely abolish EBV epithelial entry, suggesting that additional molecules are involved [26]. Indeed, it was reported that Ephrin receptor tyrosine kinase A2 (EphA2) works as an EBV receptor in gastric cancer epithelial cells [27, 28]. Once the virus enters the cytoplasm, the tegument proteins and virally encoded RNAs are released. The viral genome enters the nucleus, circularizes and remains as an intranuclear episome [29]. In the nucleus, the virus tethers to the host genome through the Epstein-Barr Nuclear Antigen (EBNA1) [30], which in turn, is involved in DNA replication by the host DNA polymerase through binding to the OriP site during latency. In general, multicopies of the EBV genome are heterochromatinized by the host cell machinery [29]. The BNRF1 viral protein binds to DAXX protein forming a complex to suppress transcription through histone methylation [31]. Besides, *BZLF1* transcripts packaged into the virions are immediately translated (1,5 h after infection) to initiate the pre-latent abortive lytic phase of infection. During this phase, the classical latency genes are expressed: Epstein-Barr nuclear antigens (*EBNAs*), latent membrane proteins (*LMPs*), viral non-coding RNAs and microRNAs, and some lytic genes such as *BZLF1* and *BRLF1*. This transient phase ends after 1- 2 weeks and virion generation does not occur [32]. Then, according to the expression profile, EBV establishes four types of latency in a cell type- and phenotype-dependent manner. Latency 0 is established in periphery resting B cells (EBERs and BARTs); latency I is established in periphery dividing B cells and Burkitt’s lymphoma (EBERs, BARTs and EBNA1); latency II is established in B cells from tonsil germinal center and Hodgkin´s lymphoma (EBERs, BARTs, EBNA1, LMP1, LPM2A, and LMP2B) and finally, latency III is established in tonsil naïve B cells and immunoblastic lymphoma (EBERs, BARTs, LMP1, LMP2A, LMP2B, EBNA1, EBNA-LP, EBNA2, EBNA3A, EBNA3B and EBNA3C) [33–35]. Latency I/II is clearly detected in tumor epithelial cells, including GC and NPC [36, 37].

Factors related to the differentiation of B lymphocytes or epithelial cells are strongly related to activation of the lytic cycle. This is a highly regulated process currently divided into three phases: Immediate-early (IE), early (E), and late (L). The switch from latency to lytic cycle involves the expression of the *BZLF1* and *BRLF1* genes, regulated by the Zp and Rp promoters, respectively [32, 38]. The encoded products, Zta and Rta proteins, are transcription factors that regulate EBV early lytic cascade. It has been suggested that Blimp1, expressed during terminal differentiation of epithelial cells, is important for Zp promoter activation, enabling lytic cycle induction [39]. Additionally, chemicals such as 12-O-tetradecanoylphorbol-13-acetate (TPA), sodium butyrate and calcium ionophores induce the EBV lytic cycle, whereas epigenetic modifications such as DNA methylation and histone deacetylation are related to inhibition of IE gene transcription [40]. In any case, the expression of both Zta and Rta proteins is always required for subsequent expression of lytic early proteins [41] such as BMRF1, SM, BHLF1, and BHRF1. Additionally, viral DNA polymerase (BALF5) [42], the DNA polymerase processivity factor (BMRF1) [43], the viral helicase (BBLF4) [44], and viral primase (BSLF1) [44], among others are currently expressed at this stage. BMRF1 and BRRF1 are transcription factors that activate the oriLyt (Lytic replication origin). The oriLyt has a complex structure containing multiple regions required for DNA replication which is executed by the viral BALF5 DNA polymerase [45]. The viral DNA replication occurs through a rolling circle-mechanism leading to the formation of concatemers which are finally cleaved and packaged [46, 47]. Once the viral DNA is replicated, late lytic genes are expressed, but the manner in which EBV late promoters are regulated is poorly understood. The late genes encode for structural proteins including nucleocapsid and glycoproteins on the viral envelope (gp350/220, gp85, gp42, and gp25). In oral epithelial cells, both late gene expression and viral maturation (lytic cycle) occur in the upper differentiated layer of stratified epithelia [17].

## Role of EBV in epithelial and lymphoid cancers

### Nasopharyngeal carcinomas

NPC is a rare type of head and neck cancer arising in the nasopharynx [48] and frequently occurring in certain populations from Central Africa or Asia [49]. In all likelihood, factors related to lifestyle or sociodemographic features are relevant in the development of this tumor [48, 49]. There are three histological types: type 1: squamous cell carcinoma; type 2: non-keratinizing carcinoma; and type 3: undifferentiated carcinoma [50]. Nearly 100% of undifferentiated NPCs, the most frequent histological type, harbor episomal copies of EBV, suggesting that this viral infection is a necessary condition for its development [9]. EBV is able to regulate multiple signaling pathways which include NF-κB, PI3K/Akt/mTOR, Wnt/β-catenin and JAK/STAT for NPC development (reviewed in [51]). In addition, EBV directly inhibits some tumor suppressors including p53 [52, 53], while indirectly reducing the expression of other tumor suppressor genes by promoting hypermethylation [54, 55]. Although EBV establishes a latency II type in NPC, abortive lytic reactivation has been detected, suggesting that early gene activation from the Zp promoter is relevant for nasopharyngeal carcinogenesis [56, 57]. In particular, EBNA1 enhances the activator protein-1 (AP-1) pathway promoting angiogenesis [58], and the expression of the zinc finger E-box binding homeobox 1 (*ZEB1*) and *ZEB2* genes, promoting EMT in NPC cells [59]. Additionally, LMP1 activates NTRK2-mediated AKT/ERK signaling pathway promoting EMT [60]. Cai et al. (2015) also demonstrated the capacity of EBV-miR-BART7-3p to promote EMT, migration and metastasis in NPC cells [61]. LMP1 increases angiogenesis by promoting VEGF expression through the JNKs/c-Jun signaling pathway [62]. Moreover, LMP1 induces the binding of NF-κB p65 to the human telomerase reverse transcriptase (hTERT) and the subsequent translocation of these proteins from the cytoplasm to the nucleus [63]. LMP1 also induces the expression of the programmed death ligand-1 (PDL-1) in NPC cells, which plays an important role in immune evasion [64]. BARF1 was able to cooperate with H-ras for inducing anchorage-independent growth as well as tumor formation in nude mice [65]. Furthermore, the EBV-encoded mi-RNAs (miR)-BART19-3p decreased apoptosis in NPC cells [66], while miR-BART5 diminished the mRNA level of the proapoptotic PUMA [67]. Similarly, EBV-miR-BART8-3p promotes NPC cell migration and metastasis via NF-κB and ERK1/2 pathways [68].

### Gastric cancer

GC is the third leading cause of cancer-related deaths worldwide and ranks fifth in relation to number of cases per year [69]. Its development is believed to be the result of a complex interaction between environmental, genetic and nutritional factors [70]. Among high-risk factors, *H. pylori* and EBV infection, tobacco smoking, alcohol consumption, and low intake of fruits and vegetables are considered to play a key role in GC development [71]. In 1990, Burke and co-workers reported the association between EBV and GC with lymphoepithelioma-like histology for the first time [72]. Shortly after, it was also demonstrated that genomic sequences of EBV were present in a subset of typical gastric adenocarcinoma [73]. Multiple EBV genomes were present in the GC cells, based on the intensity of DNA by *in situ* hybridization (ISH) signals and PCR dilution studies [73]. Epstein–Barr virus-encoded small RNAs (EBERs) are the most abundant RNAs present in infected cells and interact with several host proteins to form ribonucleoproteins complex (RNP). The prevalence of Epstein-Barr virus (EBV)-associated gastric carcinoma (EBVaGC) is between 5.0-17.9% worldwide [74], although Latin America shows a high incidence of EBVaGC [10]. It has been found that the *LMP1* oncogene, overexpressed in all EBV-associated lymphomas, is poorly or non-expressed in EBVaGC. The main viral protein whose expression is retained during EBV latency is EBNA1, which has been characterized as an oncoprotein [75]. In addition, BARF1 is expressed in the majority of EBVaGC cases [76].

### Hodgkin lymphoma

Hodgkin lymphoma (HL) or Hodgkin's disease accounts for up to 20% of all lymphomas depending on demographic factors [77–79]. HL is composed of two pathological types: Classic HL and nodular lymphocyte predominant HL (NLPHL) which show different etiopathogenesis [80]. HL is heterogeneous being composed by mononuclear Hodgkin cells, Reed-Stemberg (RS) cells, with an inflammatory infiltrate composed by B/T cells, plasma cells, histiocytes, neutrophils, eosinophils and mast cells [81]. The prevalence of EBV in HL shows sociodemographic differences [82, 83], although evidence suggests that age of primary EBV infection and acute IM are risk factors for this malignancy [80, 83].

### Burkitt lymphoma

Burkitt Lymphoma (BL) is an aggressive non-Hodgkin lymphoma (NHL) affecting B cells. Although the oncogenic mechanisms are unclear, BL is characterized by c-MYC gene translocation on chromosome 8 in a very high percentage of cases. This alteration leads to c-Myc protein overexpression [84]. Besides, this lymphoma is associated with EBV and HIV infection. In view of the involvement of different etiological factors, BL is classified as endemic (eBL), sporadic (sBL), and immunodeficiency-related [85]. The association of BL with EBV infection is widely reported. In fact, EBV was first identified in a BL sample of a patient from the equatorial Africa in the 1960s. Since then, different studies have established the clear association of EBV and sociodemographic, environmental and geographical factors in BL etiopathogenesis [86]. Early on, it was observed that areas with high incidence of endemic cases of BL in Sub-Saharan Africa and Papua New Guinea exhibited a striking overlap with areas with holoendemic malaria [85, 87]. Importantly, 95% of eBL cases in these regions are associated with EBV infection. Moreover, the average age of diagnosis is under 7 years, making eBL a leading cause of childhood malignancies in these parts of the world. On the other hand, sBL exhibits a significantly different incidence profile. It is the most common variant in Northern Africa, Europe, and North America. In these regions, this tumor is still common in children, accounting for almost one-third of all pediatric lymphomas. These lymphomas correspond to 1% to 5% of all NHL in adults [88, 89] representing a substantial proportion of all sBL diagnosed [89, 90]. The median age of incidence of sBL is 30 years [91] with peaks of incidence reported at 10 and 70 years of age [89, 92]. Another important characteristic of sBL is that only 20% to 40% of the cases are positive for EBV DNA [93]. Finally, immunodeficiency-associated BL, the third BL clinical category, is diagnosed in immunosuppressed patients either infected by HIV or transplant organ recipients [93]. The data briefly presented above highlight the complex interactions between genetic, geographical, social, environmental, and viral factors that cooperate in the development of BL. An in-depth discussion of these factors, the cellular signaling pathways involved, the viral genes involved in evasion and cell transformation exceeds the scope of this review. To obtain insights into the mechanisms involved the reader is referred to some excellent studies [34, 94–97].

### Lymphoepithelial carcinoma

Lymphoepithelial carcinoma (LEC), also called lymphoepithelioma-like carcinoma, is a rare high-grade tumor characterized by an undifferentiated carcinoma, accompanied by a non-neoplastic lymphoplasmacytic infiltrate [98]. LECs are considered as non-keratinizing squamous cell carcinomas similar to NPC (undifferentiated type) that arise in locations other than the nasopharynx [98, 99]. LEC commonly develops in the head and neck regions [100, 101], but are also diagnosed in other organs with epithelial lining [102–104]. EBV infection was detected in 87.5% of head and neck LEC [100]. Of note, this virus was found in 96.1% of the salivary gland LEC [101]. In pulmonary LEC, EBV infection was evidenced in up to 93.8% of samples and it was significantly increased when compared with non-LEC [102]. Similarly, the expression of EBV was found in 86.4% of gastric LEC, which was also significantly higher compared with the non-LEC group [103].

### EBV in other epithelial tumors

Cervical cancer is the fourth most commonly diagnosed tumor in women and the fourth cause of cancer-related death for this gender [105]. The etiological association of HPV infection with cervical cancer is well established [106]. However, less than 5% of infected women develop this disease [107], suggesting that other factors (e.g. TS, use of oral contraceptives, viral coinfection) are involved in cervical carcinogenesis. In this regard, HPV/EBV co-presence was found in epithelial cells from cervical carcinomas, ranging from 27.8% to 100%. Moreover, the frequency of EBV infection increased with the grade of the cervical lesion [108]. In cervical cancer, the expression of LMP1 and EBNA2 latent proteins was evidenced [109], which is consistent with the establishment of latency III program. Interestingly, the expression of LMP1 and EBNA1 latent proteins accompanied by BARF1 was also detected in cervical cancer [110]. However, the role of EBV in the development and/or progression of cervical tumors is still under discussion (Reviewed in [111]).

Breast cancer ranks first in both incidence and cancer-related death in women worldwide [105]. A variety of risk factors have been related to the development of breast tumors, including but not limited to, family history, diet, hormone use, alcohol consumption, and TS [112]. Some oncogenic viruses such as HPV, EBV, human cytomegalovirus (HCMV), bovine leukemia virus (BLV), and mouse mammary tumor virus (MMTV) have been identified in breast cancer [113–116]. In particular, a meta-analysis found an overall prevalence of EBV in 26% (ranging from 0 to 78%) of breast carcinomas, which suggests a potential contribution of EBV to the development of these tumors [117]. Additionally, EBV infection of immortalized human mammary epithelial cells increased the tumor formation in NOD/SCID mice. EBV-related tumors displayed a latency II type, characterized by EBNA1 and LMP1-2B expression accompanied by *BXLF2* and *BFRF3* lytic gene expression [118]. Additionally, *EBNA1*, *LMP1*, *BZLF1*, and *BARF1* transcripts were detected in breast carcinoma samples [119]. Nonetheless, the evidence is insufficient to establish an etiological role of EBV in breast cancer (reviewed in [120]).

Colorectal carcinoma ranks third in incidence worldwide, being the second cause of cancer-related death in both genders [105]. Family history of colorectal cancer or adenomatous polyposis, increased intake of red and processed meats, obesity, alcohol use, and TS are all among the risk factors associated with this cancer [121, 122]. Additionally, oncogenic viruses such as HPV, JCPyV, BKPyV and EBV have been detected in colorectal cancers (reviewed in [123]). In particular, the frequency of EBV infection in colorectal carcinoma ranges from 0% to 46% [124]. *EBNA1* and *LMP1* gene expression was detected in 14% and 25% of colorectal carcinomas, respectively. Moreover, LMP1 protein expression was associated with grade 2 adenocarcinoma [125]. Conversely, an association between EBV DNA with CpG island methylation was not detected in colorectal carcinomas [126]. Therefore, the potential role of EBV in colorectal cancers warrants further investigation.

### EBV Abortive lytic cycle

EBV role in cancer involves restrictions of complete lytic cycle activation, several studies have reported the significance of the expression of some lytic genes. The abortive lytic cycle occurs when EBV is maintained in a latent physical status with partial expression of lytic genes. The term “abortive” means that viral maturation does not occur because structural late genes are not expressed, and thus the infected cells are not lysed as normally occurs during a complete lytic cycle. Previous reports demonstrated the expression of some EBV lytic genes in epithelial cancers and EBV-associated lymphomas [127]. Zta protein was expressed in 100% nasopharyngeal carcinomas, suggesting that this IE protein is relevant in this malignancy [128]. Other studies confirmed this finding as well as the coexistence of latent and lytic gene expression in NPC [56, 129]. What´s more, there is solid experimental evidence that the EBV abortive lytic cycle is important for carcinogenesis [127, 130, 131]. For instance, knock-out mice for *BZLF1* (encoding for Zta protein) and *BRLF1* (encoding for Rta protein) genes are unable to efficiently induce lymphoproliferative disease, demonstrating that lytic genes are involved in these malignancies [132]. Moreover, an EBV mutant with enhanced Zta expression causes abortive lytic infection and lymphomas in a humanized mouse model [133]. Regarding epithelial cancers, it was observed that EBV lytic reactivation induced by TPA and sodium n-butyrate is critical for promoting genomic instability, thereby increasing the tumorigenic potential [134]. Importantly, the lytic viral protein BGLF5 was found to have the most significant effect in promoting DNA damage, genomic instability, increased cell migration, and repression of host repair-related enzymes [135]. Furthermore, expression of BALF3, the product of an EBV late gene, increased genomics instability and tumor properties of NPC cells [136]. However, the factors and mechanisms leading to abortive lytic cycle establishment are unclear.

## Xenobiotics in EBV-driven cancers

### Tobacco smoke increase EBV antibodies suggesting an increased viral load

**Fig. 1:**
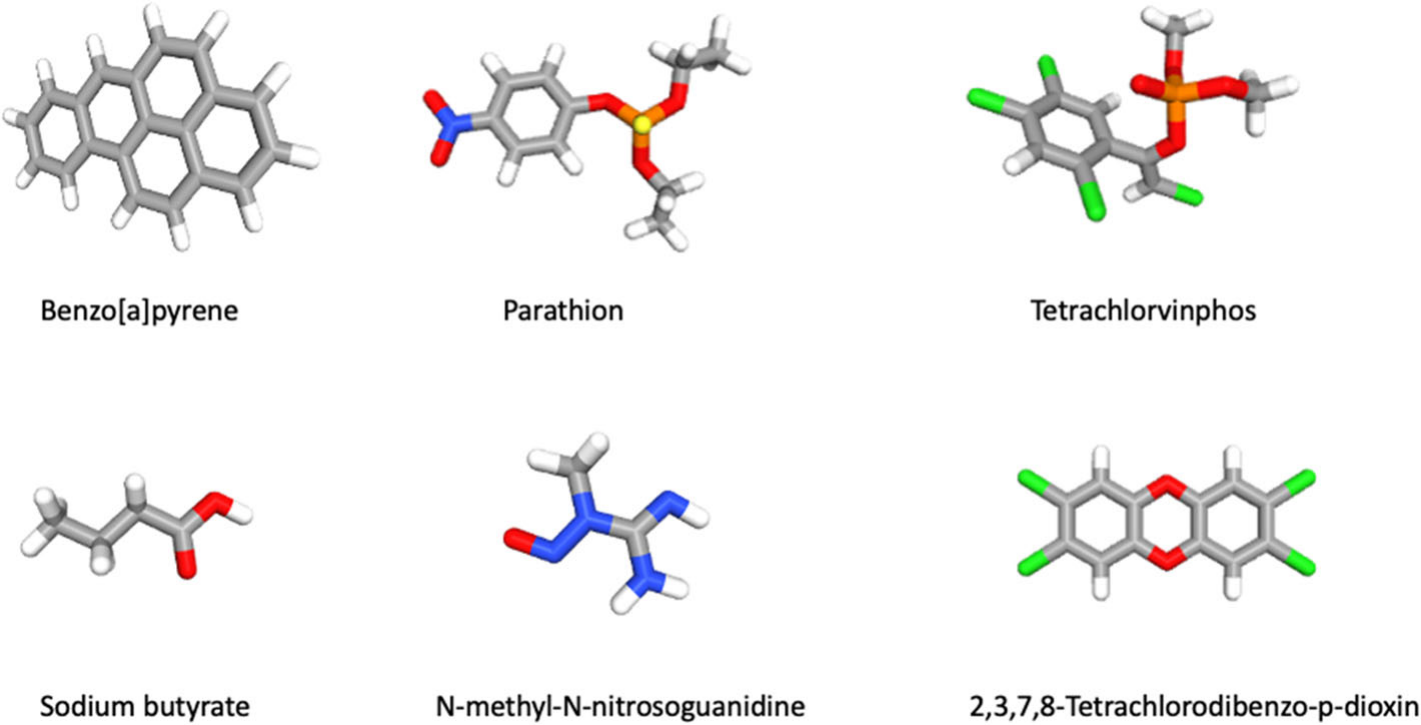
Environmental xenobiotics structure. Tobacco smoke component: benzo (a) pyrene; pesticides: Parathion, tetrachlorvinphos; Food chemicals: sodium butyrate, N-methyl-N-nitrosoguanidine and, 2,3,7,8-tetrachloroddibenzo-p-dioxin.

The International Agency for Research on Cancer (IARC, Lyon, France) has declared TS as a class 1 carcinogen in humans [137]. Extensive studies addressing molecular mechanisms and signaling pathways involved in epithelial carcinogenesis associated to TS (i.e., lung, oral cavity, esophagus, etc.) have been previously published [138–140]. More than 4,500 chemical compounds have been identified in TS, 73 of which have demonstrated carcinogenic potential [141]. These include aromatic polycyclic hydrocarbons such as benzo [α] pyrene (BaP), N-nitrosamines, benzene, aldehydes, and 1,3-butadiene, among others (Figure 1). Previous epidemiological and experimental studies have shown complex and multi-level interactions between TS components and some viral infections. For example, TS is a cofactor in cervical carcinogenesis induced by human papillomavirus (HPV) (reviewed in [111]). Cooperation between TS and Hepatitis B virus [142] or Hepatitis C virus for developing liver cancer, has also been suggested [143].

Early studies demonstrated that tobacco-derived nitrosamines requiring metabolic activation N'-nitrosonor nicotine (NNN) and 4-[methylnitrosamino]-l-[3-pyridyl]-1- butanone (NNK) increased both EBV-induced lymphocyte transformation and EBV genomes load [12]. Interestingly, epidemiological studies have suggested that TS is involved as a cofactor in EBV-mediated cancers, including B cell lymphomas, GCs, and NPCs [144–146]. Indeed, Camargo and co-workers (2014) demonstrated that smoking association with GC is higher for EBV-positive than for EBV-negative tumors [144]. The authors suggested that the interaction of smoking with EBV-positive GC may occur by EBV reactivation from latency, as occur in EBV-positives cell lines Akata and B95-8 [147]. However, additional mechanisms may be involved, and we cannot deny the possibility of additive effects of smoking with EBV. Other studies have reported positive associations between TS exposure and risk of EBV-related Hodgkin lymphoma in women from the United States [148, 149]. Additionally, some reports found a positive relationship between smoking and NPC risk [150–152] which involved elevated levels of EBV antibodies in NPC patients [153–155]. A recent study described an association between serum cotinine levels and positivity for EBV serological markers in healthy men from China [156]. Another study found that TS promotes EBV replication with viral maturation (increased gp350, a lytic late protein) in lymphoblastic cells [147]. Taken together, these findings suggest that TS components promote EBV lytic reactivation with increased viral load and dissemination [156]. Conversely, no association between EBV VCA detection, DNA viral load or EBV serology and smoking status in a cohort of 313 male subjects was reported, suggesting that TS is not related to viral lytic replication in the nasopharynx [157]. Therefore, additional studies are necessary to better understand the potential role of TS in the EBV replicative cycle.

As previously stated, EBV establishes a lytic cycle in normal epithelial cells [18, 20]. It has been hypothesized that previous genetic alterations are required for promoting EBV genome maintenance and latency establishment in epithelial cells [158, 159]. In fact, TS components themselves promote DNA damage and activation of DNA damage response (DDR) [160, 161]. Additionally, Cyclin D1 overexpression is frequently found in EBV-infected dysplastic nasopharyngeal epithelial tissues, promoting cell proliferation and clonal expansion [162]. Moreover, Cyclin D1 overexpression can override growth inhibition and cellular senescence promoted by EBV infection [162, 163]. The same authors previously reported that *CDKN2A* (p16) gene loss through homozygous deletion is a frequent alteration in NPC [164]. Interestingly, TS compounds promote these molecular alterations in different cellular models and smoker subjects, suggesting the possibility that TS may be involved in promoting the EBV genome maintenance and consequently, the latency establishment in epithelial cells [165–169]. This is an interesting possibility to be addressed in future studies. Taken together, it is plausible that TS compounds are directly involved in long-term EBV propagation in epithelial cells, with a role in cancer initiation. Additional experimental approaches are needed to dissect the specific role of TS in EBV-associated carcinogenesis.

### Pesticides

Many studies have evaluated the relationship between pesticide exposure and the development of diverse hematological (i.e., lymphoma) or epithelial (i.e., breast) tumors [170–173]. In fact, the IARC classified malathion and diazinon as “probably carcinogenic” (group 2A), and parathion and tetrachlorvinphos as “possibly carcinogenic” (group 2B) (Figure 1) [174]. In particular, organophosphorous pesticides like parathion and malathion have been used extensively to control mosquito plagues and a wide range of sucking and chewing pests that target field crops, fruits, and vegetables. It has been considered that reactive oxygen species (ROS) are caused by such substances which may be involved in the toxicity of various pesticides. Studies have shown that catechol formation is a risk factor for breast cancer since it gives rise to quinones that cause DNA damage and generate ROS which promote oxidative damage [175]. Some organophosphate insecticides are also used as herbicides, the most successful example is glyphosate, which is consumed by humans through food products and can be detected in urine, serum, and breast milk samples. It is considered dangerous to the population according to IARC (2015) which declared it as “possibly carcinogenic” [176]. However, studies evaluating a potential relationship between pesticide/herbicide exposure and EBV infection in cancer, are scarce. Of note, it was reported that chlorpyrifos (CPF), a common organophosphate (OP) pesticide, can promote EBV lytic cycle activation through OS in lymphoblastic cells. Besides, lytic cycle activation occurred by increasing BZLF1 levels in a CPF dose-dependent manner [177]. Organochlorines, frequently used as pesticides, enhance the expression of EBV early antigens (EA), promoting lytic infection and increasing the risk of NHL [178–180]. In addition, an increased risk of hairy cell leukemia (HCL) in an organochlorine dose dependent-manner was found in subjects with increased EBV EA IgG, suggesting a functional cooperation between EBV and organochlorines [181]. Moreover, extranodal natural killer (NK)/T-cell lymphoma nasal type (NNKTCL), which is etiologically associated with EBV infection [182–185], was found to be related to pesticides and exposure to some chemical solvents, which suggests the possibility of EBV/pesticide cooperation in this malignancy [186].

As previously mentioned, EBV infection has been implicated in 95% of eBL cases from high-risk regions and less than 30% from low-risk regions (sBL) [187]. The role of pesticides in pediatric eBL and sBL is not clear. Only some studies addressed pesticide exposure as a risk factor for BL or HL and established associations with age or demography. One study reported that, pesticide use was associated with increased risk of developing childhood hematological diseases, including HL and NHL [188]. Additionally, Latifobic L et al (2020) reported that association between pesticide exposure and HL was higher in subjects less than 40 years [189]. Interestingly, maternal exposure to insecticides in France (OR 1.6, 1.3-2.1, 95%IC) was related to an increased risk of NHL and HL during childhood [190]. Conversely, one study in Great Britain did not support the notion that parental pesticides or solvent hydrocarbon exposure increase the risk of childhood lymphoma [191], and no evidence of elevated risk associated with residential proximity at birth to agricultural use land for most childhood cancers was observed in Texas, US [192].

Taken together, considering experimental and epidemiological findings, it is possible to speculate a potential role of pesticides in the promotion of EBV reactivation during leukemia/lymphoma development.

### Environmental pollutants

**Fig. 2:**
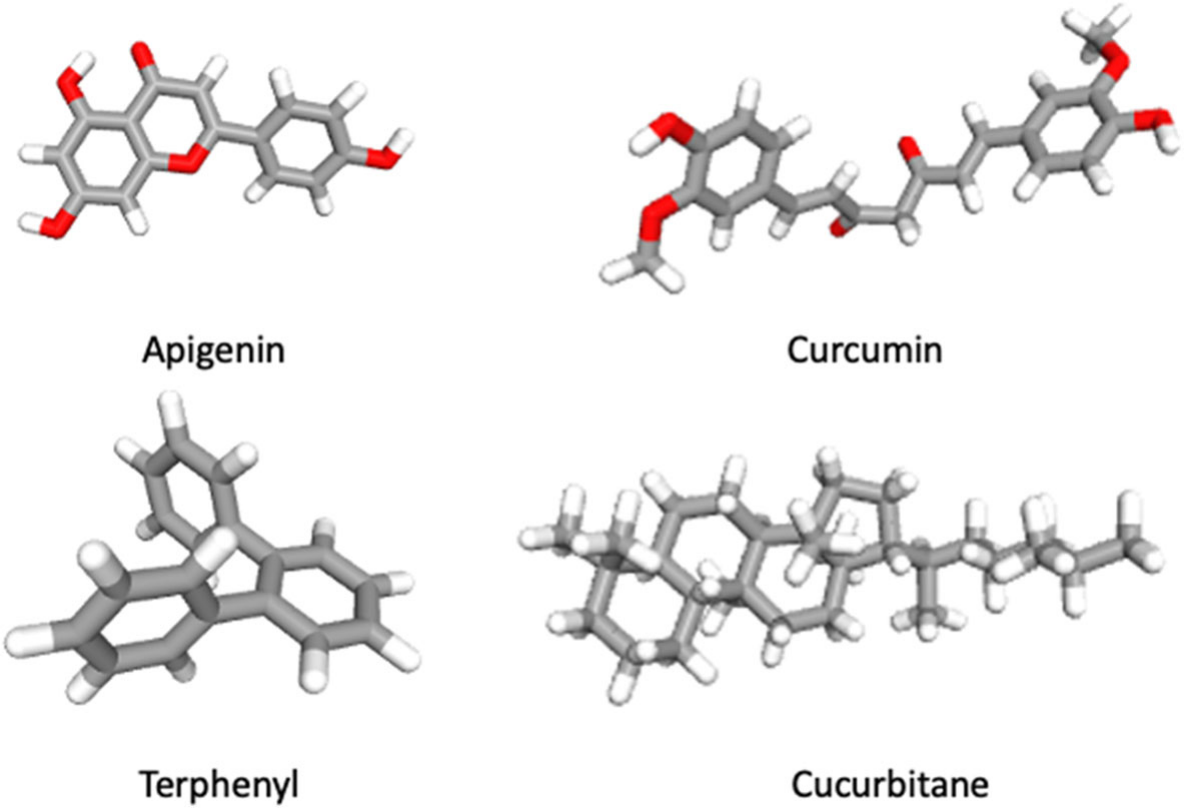
Structure of natural compounds (Apigenin, curcumin, terphenyl and cucurbitane) with inhibitory effects in EBV replication and EBV-driven cell proliferation.

It has been determined that high concentrations of persistent organic pollutants (POP) such as polychlorinated biphenyls (PCB), hexachlorobenzene (HCB), and some chlordanes (Figure 2) were associated with increased IgG antibodies to EBV early antigens in patients with NHL. These findings suggest the possibility that POP exposure might be related to EBV reactivation in NHL patients with a subsequent increase in EBV antibodies [193]. Furthermore, it was shown that prolonged exposure to some chemical compounds such as H2S, NH3, SO2, HF, NO2, chlorides, nitroesters, and dust significantly increased EBV IgG/IgM antibodies in chemical factory employees when compared with non-exposed blood donors. Moreover, enhanced immunological disturbances and hepatopathies were observed in the group of exposed subjects [194]. Thus, these findings suggest an increase in EBV lytic activation through persistent environmental contaminants.

### Food chemicals

Dioxins are persistent contaminant polyhalogenated dibenzo-p-dioxins such as 2,3,7,8-tetrachlorodibenzo-p-dioxin (TCDD) from diverse environmental sources with bioaccumulation in the food chain (Figure 1) [195]. Approximately 90% of dioxins-like compound exposure occurs through ingestion of contaminated food, such as fish, meat, eggs, or milk, among others [196, 197]. In 1976, after an industrial accident in Italy in which workers were acutely exposed to TCDD, a high frequency of epithelial and lymphoid cancers was observed [198]. Mechanistically, there is evidence that activation of the aryl hydrocarbon receptor (AhR) upon TCDD binding leads to *BZLF1* gene expression and EBV reactivation in both B cells and salivary epithelial cells, which shows potential consequences in autoimmune diseases and cancer [199]. In fact, canonical AhR activation involves binding to lipophilic agonists (xenobiotics) which diffuse through the plasma membrane, subsequently binding to AhR nuclear translocator protein (Arnt) with the complex being translocated to the nucleus for DNA binding. The AhR/Arnt complex binds the xenobiotic-response elements (XRE) located in the promoter regions of several genes and modulate their expression [200]. In particular, Phase I (CYP1A1) and Phase II (UGT1A1) enzymes are expressed upon XRE activation [201]. Additionally, it has been established that activated AhR modulates the immune response to viral infections, which varies in a cell type- and pathogen-dependent manner [202–204]. Therefore, it is possible to speculate that such alterations may be involved in the dioxin-mediated induction of EBV lytic activation and cancer development. It was also found that EBNA3, expressed during latency III in B cells, directly binds to AhR for dioxin-response element (DRE) activation. Interestingly, the presence of TCDD stabilized such EBNA3/AhR interaction. Since EBNA3 counteracts TCDD-induced cell growth inhibition in EBV-infected B cells, it is suggested that EBV uses EBNA3/AhR to oppose the negative effects of dioxin/AhR interaction on cell proliferation and survival [205].

Food chemicals such as volatile N-nitrosamines and precursors as well as host-related factors are also associated with NPC development [206]. Volatile N-nitrosamines are present in foodstuffs (i.e., Chinese salted fish) and preserved food from areas with a high prevalence of NPC [207–209]. N-methyl-N′-nitro-N-nitrosoguanidine (MNNG, a nitrosamide) was reported to increase EBV reactivation (Figure 1) [210]. In addition, a mixture of MNNG, 12-O-tetradecanoylphorbol-13-acetate (TPA) and sodium butyrate significantly increased the genomic instability and invasiveness of EBV positive NPC cells, suggesting cooperation between compounds for EBV-mediated carcinogenesis [210]. Different studies have also confirmed an increased risk of NPC in North Africa associated with rancid butter, rancid sheep fat, and quaddid consumption. For instance, a role was suggested for butyric acid, which is released by hydrolysis from glyceride when butter becomes rancid [211]. Furthermore, it was demonstrated that aflatoxin B1 (AFTB1), a mycotoxin present in some agricultural crops (i.e., wheat, maize, and nuts), increased EBV-mediated PBL transformation and EBV genomes per cell [12]. Accordingly, Accardi and co-workers (2015) showed that AFTB1 increased EBV replication and viral load in primary and immortalized B cells. Moreover, it was discovered that AFTB1-mediated *BZLF1* expression requires phosphatidylinositol 3-kinase (PI3K) signaling pathway activation [212]. Conversely, incubation with N-acetylcysteine, an oxygen-free radical scavenger, reduced EBV reactivation, demonstrating the role of OS in promoting the EBV lytic cycle. In addition, curcumin, a known antioxidant polyphenol derived from *Curcuma longa* with antitumor and antioxidant properties, inhibited the proliferation of EBV-positive NPC cells by decreasing the expression of EBNA1 [213]. In fact, there is evidence that this natural compound rescues tumor cells from epithelial-mesenchymal transition (EMT), suggesting a potential therapeutic utility [214, 215]. Other natural compounds such as cucurbitane glycosides from the fruits of *Siraitia grosvenorii* (monk fruit) showed inhibitory effects on EBV reactivation (Figure 2). Importantly, the flavonoid apigenin suppressed the activity of the EBV Zp and Rp promoters, as demonstrated by reporter assays. In particular, a region between -134 to -51 within the Zp for Sp1/Sp3, MEF2D, ATF-1, ATF-2 and CREB binding, was required for apigenin inhibition [216]. Thus, apigenin may be a potential dietary compound for prevention of EBV reactivation.

## Conclusions

**Fig. 3:**
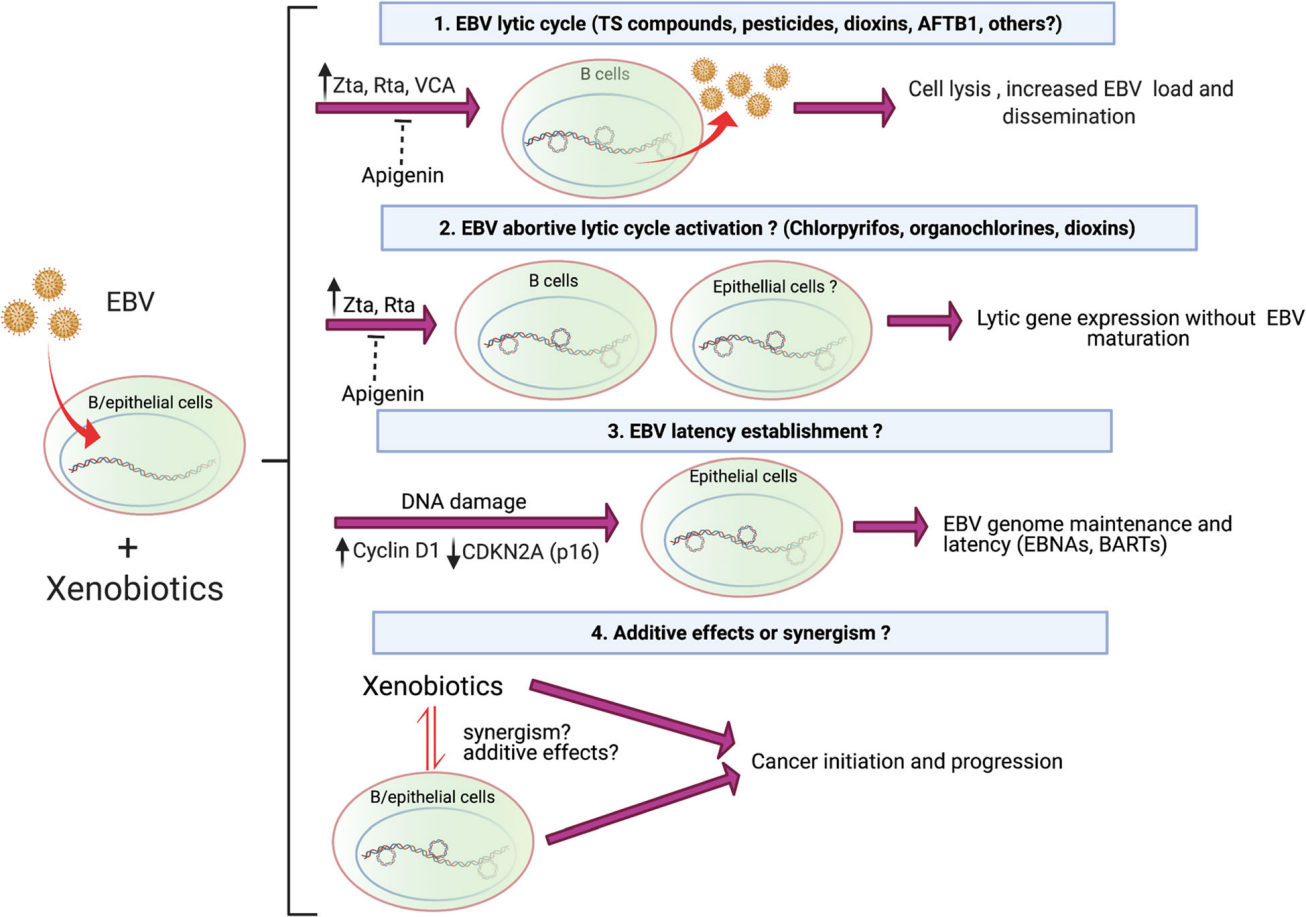
A suggested model in which environmental xenobiotics are involved in EBV-mediated cancers: 1. Xenobiotics promote EBV lytic cycle leading to cell lysis and virus dissemination from B cells; 2. Xenobiotics promote the abortive lytic infection, which involves the expression of some latent and lytic genes although without viral maturation; 3. Xenobiotics promote DNA damage which can lead to mutations facilitating EBV genome maintenance and latency in epithelial cells; 4. EBV and xenobiotics promote synergism or additive effects for cancer initiation and progression. Created with BioRender.com.

Xenobiotics such as those present in tobacco smoke, pollution, pesticides, and food affect the EBV replicative cycle, which can lead to cancer. Such a functional interaction could occur at different levels. First, xenobiotics are involved in EBV lytic activation leading to viral dissemination from B cells. Second, xenobiotics are involved in EBV lytic activation leading to the occurrence of abortive lytic infection, in turn promoting both lymphoid and epithelial cancer progression. Third, xenobiotics that promote OS and DNA damage could facilitate EBV genome maintenance and the establishment of latency in epithelial cells during cancer initiation. However, additional studies are warranted to confirm this possibility. Fourth, additive or synergistic effects in EBV-driven cancers with xenobiotics involved in cancer development are plausible. Therefore, considering the current knowledge about EBV/environmental xenobiotic interaction, a general model of carcinogenesis is shown in Figure 3. We cannot deny the possibility that additional mechanisms of EBV/xenobiotic interaction may be involved in carcinogenesis. Therefore, more studies are warranted to dissect such mechanisms and signaling pathways involved in EBV gene expression alterations and their interaction with xenobiotics. This knowledge will impact both prevention and therapeutic possibilities for EBV-associated cancers.

## Data Availability

Not applicable
